# *OsARF11* Promotes Growth, Meristem, Seed, and Vein Formation during Rice Plant Development

**DOI:** 10.3390/ijms22084089

**Published:** 2021-04-15

**Authors:** Katherine Sims, Fatemeh Abedi-Samakush, Nicole Szulc, Monika Gyongyi Macias Honti, Jim Mattsson

**Affiliations:** 1 Department of Biological Sciences, Simon Fraser University, 8888 University Drive, Burnaby, BC V5A 1S6, Canada; Katherine_Sims@sfu.ca (K.S.); sama6365@gmail.com (F.A.-S.); nszulc@alumni.ubc.ca (N.S.); monikagyongyi.maciashonti@kaust.edu.sa (M.G.M.H.); 2 Plant Cell and Developmental Biology, King Abdullah University of Science and Technology, Ibn Al Haytham Bldg.2, Thuwal 23955-6900, Saudi Arabia

**Keywords:** auxin response factor, auxin signaling, auxin perception, meristem, vein patterning, leaf expansion, root elongation, seed development, rice fecundity

## Abstract

The plant hormone auxin acts as a mediator providing positional instructions in a range of developmental processes. Studies in *Arabidopsis thaliana* L. show that auxin acts in large part via activation of Auxin Response Factors (ARFs) that in turn regulate the expression of downstream genes. The rice (*Oryza sativa* L.) gene *OsARF11* is of interest because of its expression in developing rice organs and its high sequence similarity with *MONOPTEROS/ARF5*, a gene with prominent roles in *A. thaliana* development. We have assessed the phenotype of homozygous insertion mutants in the *OsARF11* gene and found that in relation to wildtype, *osarf11* seedlings produced fewer and shorter roots as well as shorter and less wide leaves. Leaves developed fewer veins and larger areoles. Mature *osarf11* plants had a reduced root system, fewer branches per panicle, fewer grains per panicle and fewer filled seeds. Mutants had a reduced sensitivity to auxin-mediated callus formation and inhibition of root elongation, and phenylboronic acid (PBA)-mediated inhibition of vein formation. Taken together, our results implicate *OsARF11* in auxin-mediated growth of multiple organs and leaf veins. *OsARF11* also appears to play a central role in the formation of lateral root, panicle branch, and grain meristems.

## 1. Introduction

The rice plant (*Oryza sativa* L.) is one of the most important crops in the world and serves as a staple food source for over half of the global population. There is an ongoing effort to characterize genes with functions in developmental processes that impact economically important quantitative traits such as shoot and root size, leaf width, number of panicles, seeds per panicle, and seed size. There are also efforts to engineer rice with C4 traits such as high vein density and high rate of photosynthesis in leaf vein bundle sheath cells to reduce photorespiration [1,2]. The plant hormone auxin plays a role in many of these developmental programs since it regulates cell division and expansion, meristem development, and vascular patterning [3,4,5]. In recent times, it has become clear that the formation of an embryo, leaves, and roots depend on the generation of local auxin maxima by polar auxin transport [6,7,8]. 

Auxin mediates growth and development by regulating the expression of auxin-responsive genes [9]. Although the auxin signaling pathway is simple, involved core proteins are represented by large gene families, allowing for a wide range of context-dependent cellular responses [10,11,12,13]. The main components of auxin signaling have been studied intensely in *Arabidopsis thaliana* L. and involve three protein families: the F-box TRANSPORT INHIBITOR RESPONSE 1/AUXIN SIGNALING F-BOX PROTEIN (TIR1/AFB) auxin co-receptors, the AUXIN/INDOLE-3-ACETIC ACID (AUX/IAA) transcriptional repressors, and the AUXIN RESPONSE FACTOR (ARF) transcription factors [9,14,15,16]. 

Auxin regulates the transcription of downstream genes by mediating the binding of AUX/IAA transcriptional repressors to F-box TIR1/AFB proteins in the SCFTIR1/AFB complex [9,13,17]. This interaction promotes poly-ubiquitination of AUX/IAA proteins and degradation via the 26S proteasome [12,14,15,18]. In the absence of AUX/IAA repressors, ARFs can homodimerize and activate or repress transcription by binding to auxin responsive elements (AuxREs) in target genes [16,19,20]. Both the motif binding preference and the positional relationship of repetitive AuxREs contribute to the specificity in auxin response [17,18,19]. ARF proteins can also recruit protein complexes that lead to a switch from closed to open chromatin [20], and bind transcription factors from other protein families, integrating auxin signaling with other hormones and environmental signals [21]. 

Based on loss-of-function mutants, ARF transcription factors play pivotal roles in embryo, root, leaf, flower, and vascular development in *A. thaliana* [22,23,24,25,26,27,28,29,30,31,32] and are also involved in responses to abiotic and biotic stressors [10,33,34,35]. The rice (*Oryza sativa* L.) genome contains 25 *Oryza sativa AUXIN RESPONSE FACTOR (OsARF)*-encoding genes [36,37]. Mutant phenotypes have been described for several rice *OsARF* genes. Plants expressing anti-sense *OsARF1* mRNA exhibit low vegetative growth, curled leaves, and sterility [38]. T-DNA insertional mutants of *osarf12* and *osarf16* display dwarfism, abnormal root growth and loss of phosphate and iron homeostasis [39,40,41,42]. Rice *osarf18* mutants show reduced stature, abnormal cell differentiation, and incomplete seed filling [43]. Mutants in the *OsARF24* gene have reduced height and leaf width, combined with distorted leaf phyllotaxy and flag-leaf angle [44]. Loss of *OsARF19* function results in enlarged vegetative organs and abnormal flowers [45], while overexpression indicated a role in tillering angle via regulation of *OsGH3-5* and *BRI1* [46]. Furthermore, repression of *OsARF12*, *16*, *17,* and *25* results in increased leaf inclination [47,48]. As in *A. thaliana*, microRNAs modulate levels of rice ARF transcripts [47,49,50].

The *OsARF11* gene [37] is known to be expressed at higher levels in the shoot apical meristem, developing panicles, calli, and at lower levels in developing leaves, roots, and seeds [51,52,53], suggesting that it may play a role in the development of these structures. *OsARF11* is also the closest rice homolog of the *MONOPTEROS/ARF*5 gene [37], which has prominent roles in *A. thaliana* development [9,26,31,54,55]. In this study, we assessed the function of *OsARF11* by a systematic comparison of *osarf11* insertion mutants with wildtype background plants during development. We show that the *OsARF11* gene contributes to the development of all assessed organs and leaf veins and that the mutants have reduced responses to auxins. 

## 2. Results

### 2.1. Genotype of OsARF11 Mutant Plants

Two independent mutants in the *OsARF11* gene (accession no. Os04g0664400) were obtained from the Taiwan Rice Insertional Mutant (TRIM) [49] and the TOS-17 transposon [50] populations. PCR, using primers matching the *OsARF11* gene and inserted DNA, and subsequent sequencing of amplicons (Figures S1–S3) confirmed that a T-DNA is inserted in exon 11 of the *OsARF11* open reading frame (hereafter *osarf11^TRIM^* mutant allele), while the TOS-17 line has a TOS-17 transposon inserted in the fifth exon (*osarf11^TOS-17^* mutant allele) (TRIM and TOS-17 databases). 

We observed that offspring from heterozygous insertion mutants from both alleles segregated approximately 3:1 for tall and shorter plants (data not shown). PCR-based genotyping revealed that shorter plants were homozygous for insertion (Figure S1). The shorter mutant phenotype confirmed unquantified observations by insertion mutant providers available at their websites. Mutant phenotypes were only observed in plants genotyped as homozygous for mutant alleles, consistent with recessive loss-of-function alleles. Real-Time quantitative PCR indicated that the *OsARF11* transcript spanning the insertion site was present in wildtype seedlings and absent in seedlings homozygous for mutant alleles (data not shown). While homozygous *osarf11^TRIM^* mutants showed reduced fecundacy, homozygous *osarf11^TOS-17^* mutants were completely sterile. Since *osarf11^TRIM^* mutants could be maintained as homozygous populations, we first show multiple angles of analysis of this line, followed by an analysis of traits that overlap in plants genotyped as homozygous for the *osarf11^TOS-17^* allele. 

### 2.2. Morphology of OsARF11^TRIM^ Mutant Plants

#### 2.2.1. Seedlings

**Figure 1:**
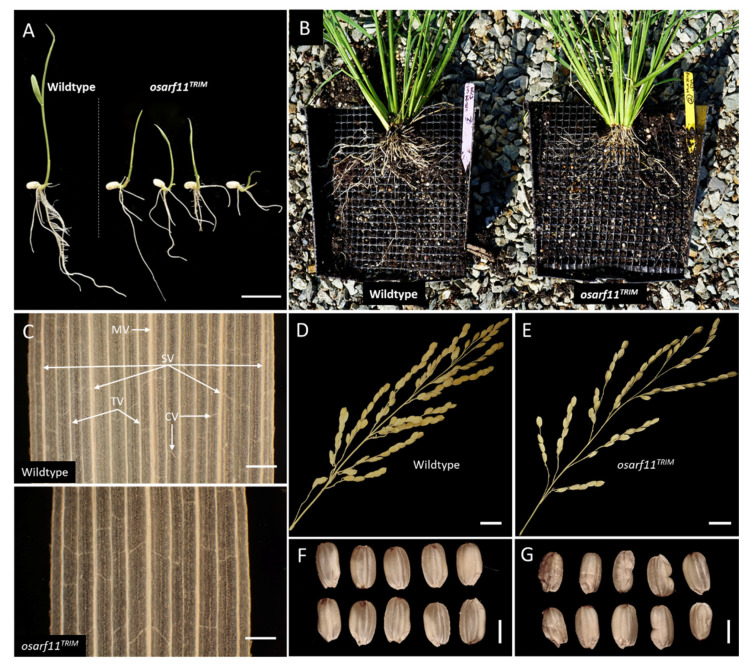
Phenotypes observed in *osarf11^TRIM^* plants. (**A**) Common shoot and root phenotypes of 10-day old *osarf11^TRIM^* seedlings. (**B**) Root growth of four-month-old adult plants. (**C**) Cleared segment, widest region of leaf blade, three-week-old wildtype (top) and *osarf11^TRIM^* (bottom) seedlings. Leaf venation classes; MV, midvein; SV, secondary vein; TV, tertiary vein; CV, commissural vein. Dried adult panicles and seeds harvested from (**D**) wildtype and (**E**) *osarf11^TRIM^* plants. Range of seed morphologies observed in (**F**) wildtype and (**G**) *osarf11^TRIM^* plants. Scale bars: (**A**) = 15 mm, (**C**) (top) = 0.3 mm, (**C**) (bottom) = 0.3 mm, (**D**) (top) and (**E**) (top) = 10 mm, (**D**) (bottom), and (**E**) (bottom) = 3 mm.

**Figure 2:**
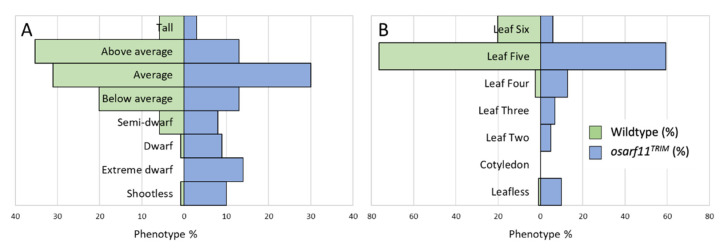
Seedling shoot size phenotype categories of 119 wildtype and 101 *osarf11^TRIM^* 14-day-old seedlings. (**A**) Shoot size groups based on the following criteria: tall > 135 mm, above average = 116–135 mm, average = 91–115 mm, below average = 66–90 mm, semi-dwarf = 41–65 mm, dwarf = 20–40 mm, extreme dwarf < 20 mm, leafless = no above ground organs. (**B**) Most recently emerged leaf primordia as observed under stereo microscope.

We repeatedly saw a range of slender and short shoots, also known as culms, companioned by dwarfed root systems in *osarf11^TRIM^* seedlings relative to corresponding wildtype background (Figure 1A). Since size variation occurs also in wildtype seedlings, we used discrete size categories to assess 119 wildtype and 101 *osarf11^TRIM^* 14-day-old seedlings (Figure 2A). The *osarf11^TRIM^* population had fewer tall and above-average height seedlings, and about the same number of average-sized seedlings relative to the wildtype population. The largest difference was in the dwarf categories, where many more mutants than wildtype were classified as having dwarf stature; 14% mutant seedlings were extreme dwarfs whereas none of the wildtype seedlings fell into this category. While 0.8% wildtype seedlings presented a shoot-less phenotype, 10% mutant seedlings showed this phenotype. When the same populations were categorized based on the most recent leaf to emerge, we saw a skewing that indicated delayed emergence in mutants (Figure 2B). In both populations, leaf five was the most common leaf to have emerged; however, the mutant population had 6% seedlings and wildtype 20% seedlings with leaf six emerged, and the mutant population had more seedlings with leaf four, three, and two as the most recently emerged. 

Two-week-old *osarf11^TRIM^* seedlings were, on average, 38% lighter than wildtype seedlings, a reduction that, when assessed in a subpopulation, was approximately evenly distributed in shoots and roots (Table S1). Shoots of *osarf11^TRIM^* seedlings were 25% shorter than wildtype shoots. At the same time, mutant leaves two, three, and four had leaf blades that were 22%, 21%, and 36% shorter than their respective wildtype blades. Mutant leaves two, three, and four also had 17%, 19%, and 21% less-wide blades compared to wildtype. Mutant plantlets displaying extreme dwarfism did not survive the transfer to clay media and were not included in adult phenotype scoring. Thereafter and throughout development, the most conspicuous and consistent phenotype of *osarf11^TRIM^* shoots was a 17–21% narrower leaf width relative to wildtype plants (Table S1). As *osarf11^TRIM^* plants matured, we found in two trials no significant difference in both plant height and shoot biomass, suggesting plants defective in *OsARF11* eventually catch up to its wildtype counterparts (Table S1). However, in a third trial, *osarf11^TRIM^* plants were significantly shorter (Figure S4), suggesting that this trait may vary with growth conditions.

#### 2.2.2. Roots

**Figure 3:**
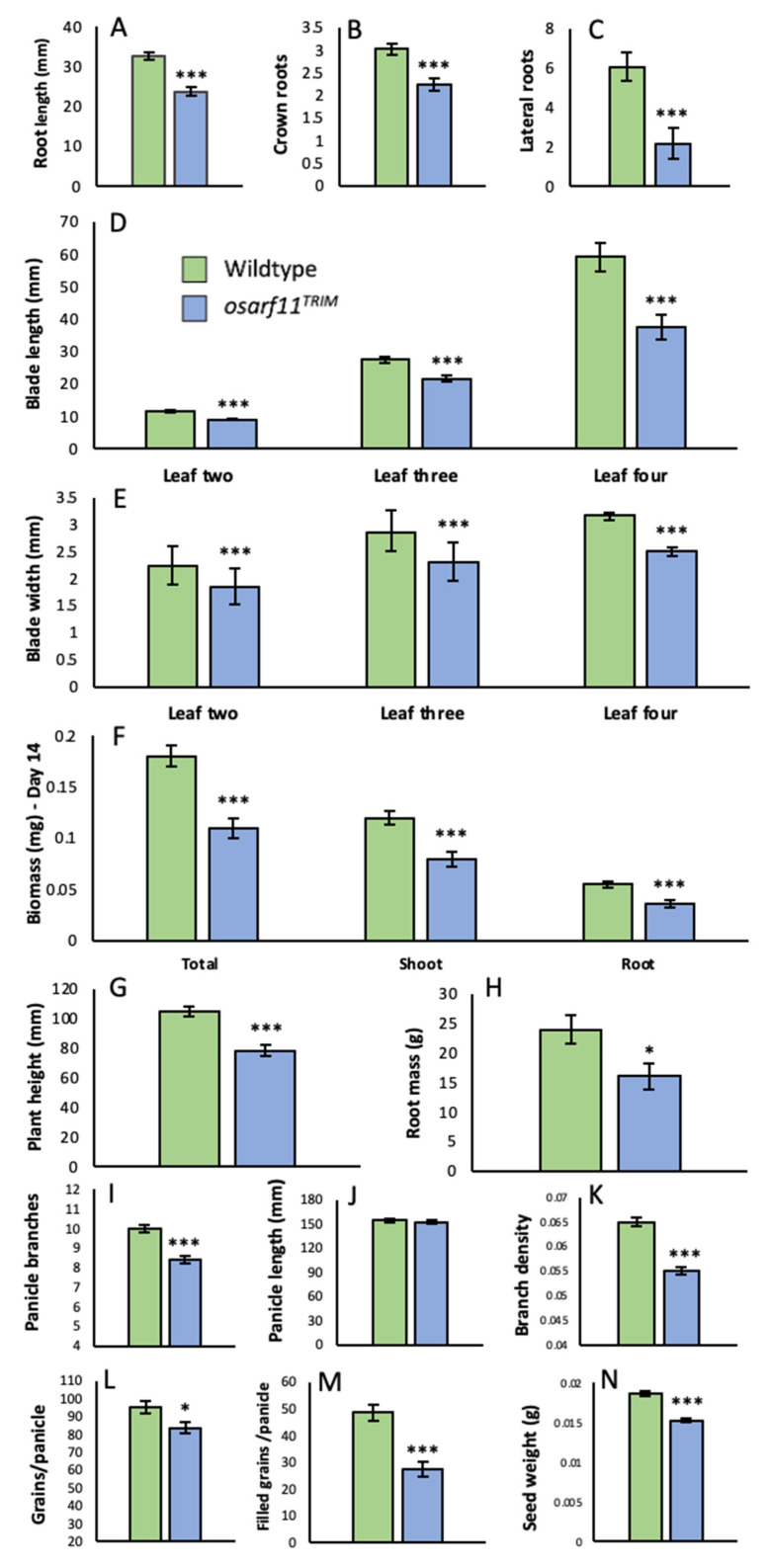
Quantitative phenotypes observed in *osarf11^TRIM^* plants. (**A**) Primary root length, (**B**) crown root number, and (**C**) lateral root number in seven-day-old seedlings. (**D**) Blade length and (**E**) blade width measured at day 14 of development in leaves two, three, and four of the primary shoots. (**F**) Plant weight and (**G**) plant height of 14-day old seedlings. (**H**) Dry root weight of six-month-old adult plants. (**I**) Panicle branch number, (**J**) panicle length, and (**K**) panicle branch density of dried adult panicles. Branch density = number of panicles/panicle length. (**L**) Total grain number harvested per panicle. (**M**) Number of filled grains per panicle. (**N**) Average seed weight. Asterisks indicate significant differences between wildtype and mutant categories (Student’s *t* test; * *p* < 0.05, *** *p* < 0.001). Error bars = standard error.

Rice has a root system that develops post-embryonically and consists of seminal, crown, and lateral roots. We compared root growth in germinating *osarf11^TRIM^* and wildtype seedlings. After one week of growth, we observed that *osarf11^TRIM^* seedlings had primary roots that were 27% shorter, had 26% fewer crown roots, and 64% fewer lateral roots relative to wildtype seedlings (Figure 3A–C). Although variable, the reduced root growth in the *osarf11^TRIM^* line remained a consistent phenotype (Figure 1B). The dried root system of adult *osarf11^TRIM^* plants weighed, on average, 33% less than that of wildtype plants (Figure 3H).

#### 2.2.3. Panicle and Seed Development

Three independent trials revealed a significant decrease in the number of lateral panicle branches along the main panicle axis (Figure 3I). Since the panicle length from first branch to the apex did not differ between wildtype and mutant plants, the reduced number of branches also translated into a reduction in branch density in *osarf11^TRIM^* plants relative to wildtype (Figure 3K). Mutant *osarf11^TRIM^* plants developed 12% fewer grains per panicle compared to wildtype (Figure 3L). When plants of *osarf11^TRIM^* and wildtype plants were grown side by side, we observed about 45% fewer filled grains per panicle in *osarf11^TRIM^* mutant plants (Figure 3M). Additionally, *osarf11^TRIM^* seeds were 17% lighter (Figure 3N), shorter, less wide, and thinner than corresponding wildtype seeds (Table S1).

### 2.3. Leaf Vascular Pattern Formation Is Altered in osarf11 Mutants

#### 2.3.1. Vein Number and Density

**Figure 4:**
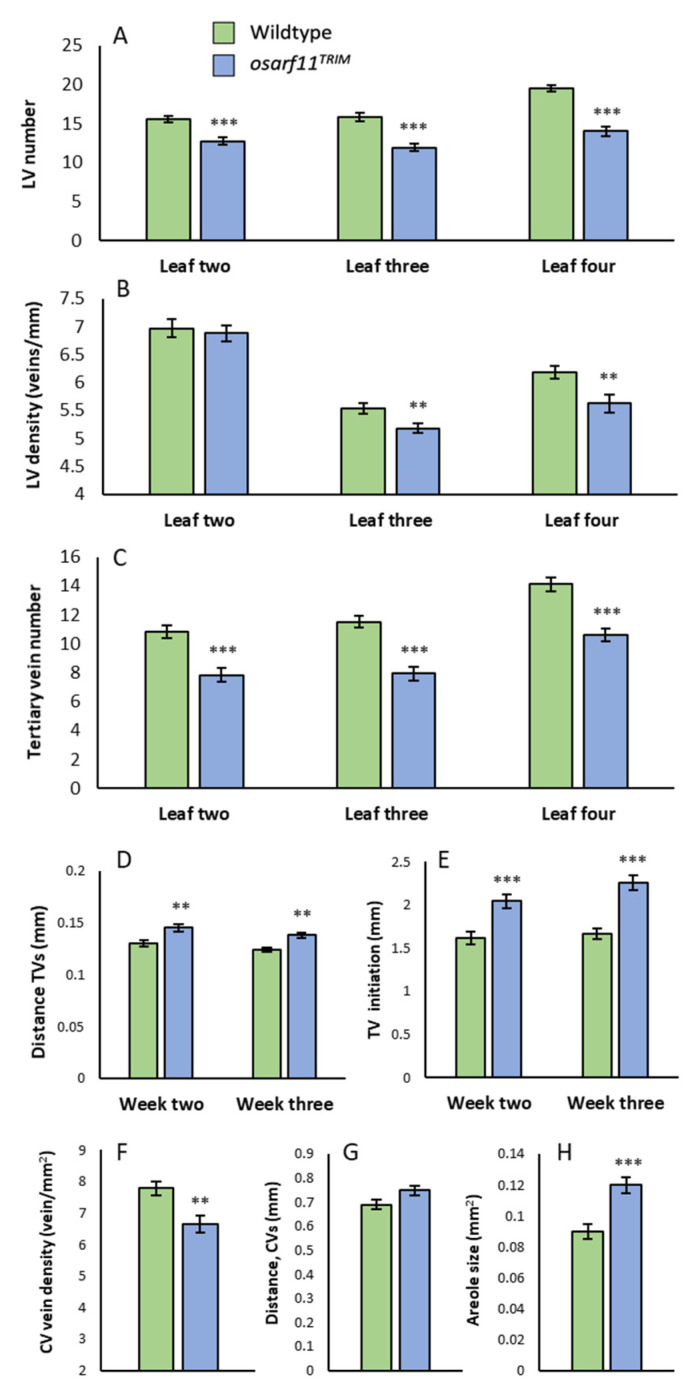
Leaf venation defects in *OsARF11^TRIM^* seedlings. (**A**) Longitudinal vein number, (**B**) longitudinal vein density, and (**C**) tertiary vein number in leaves two, three, and four of the primary shoot. (**D**) Average distance between tertiary veins, measured at the widest part of leaf four at week two and three of development. Each data point represents an average of three distances measured per leaf scored. (**E**) Tertiary vein initiation, measured from the tip, in leaf four at week two and three. (**F**) Commissural vein density; number of veins per leaf area, measured at the widest part of leaf four. (**G**) Distance between two adjacent commissural veins and (**H**) areole size in leaf four. Each data point in (**G**,**H**) represents an average of 10 measurements per leaf. Asterisks indicate significant differences with respect to wildtype (Student’s *t* test; ** *p* < 0.01, *** *p* < 0.001). Error bars = standard error. *LV*, longitudinal veins; *TV*, tertiary veins; *CV*, commissural veins.

To assess a potential role of *OsARF11* in vascular patterning, we counted longitudinal veins in cleared second, third, and fourth leaves of the primary shoot in wildtype and *osarf11^TRIM^* populations. Mutant leaves had 17–28% fewer longitudinal veins (Figure 4A). When the number of veins per mm width of the blades was calculated, we observed a reduction in vein density in leaf three and four but not two in *osarf11^TRIM^* mutants. The reduction in vein density was most obvious in developing leaves but was also present in fully grown leaves. At two weeks, *osarf11^TRIM^* showed an average 7% reduction in longitudinal vein density in the third leaf and a 9% reduction in leaf four (Figure 4B). In a separate population scored at three weeks, a reduction of 5% and 6% was detected in the third and fourth leaf, respectively (Table S2). Floral organ vasculature was also assessed, but no abnormalities were observed (data not shown).

#### 2.3.2. Tertiary Veins

In addition to the single midvein, we observed that the number of thicker secondary veins was invariable at four in the second, third, and fourth leaves of wildtype and mutant seedlings. This suggests that the difference in vein number between the two genotypes were in the number of tertiary veins. Counting of tertiary veins showed that mutant second, third, and fourth leaves had on average 27%, 33%, and 29% fewer tertiary veins than respective wildtype leaves (Figure 4C). We also directly measured the distance between tertiary veins and found it to be significantly higher in the fourth leaf of two and three-week-old mutant leaves relative to wildtype (Figure 4D). Tertiary veins evaluated in the fourth leaf were also found to initiate further from the leaf tip in *osarf11^TRIM^* compared to wildtype plants in both two and three-week-old seedlings (Figure 4E, Figure S5). 

#### 2.3.3. Commissural Veins and Areole Size

When distance between transversal commissural veins was measured directly, the average distance was highly variable. However, when the number of commissural veins were counted across the widest part of the leaf width, the results showed that the commissural vein density was 12% (2-week-old) and 15% (3-week-old) lower in the fourth leaf of *osarf11^TRIM^* mutants than corresponding wildtype leaves (Figure 4F). We also measured the area enclosed by two longitudinal veins and two commissural veins. The areole size was 27% (2-week-old; Figure 4H) and 37% (3-week-old; Table S2) larger in the fourth leaf of mutant seedlings relative to wildtype seedlings. 

### 2.4. Morphometric Analyses of OsARF11^TOS-17^ Mutants

**Figure 5:**
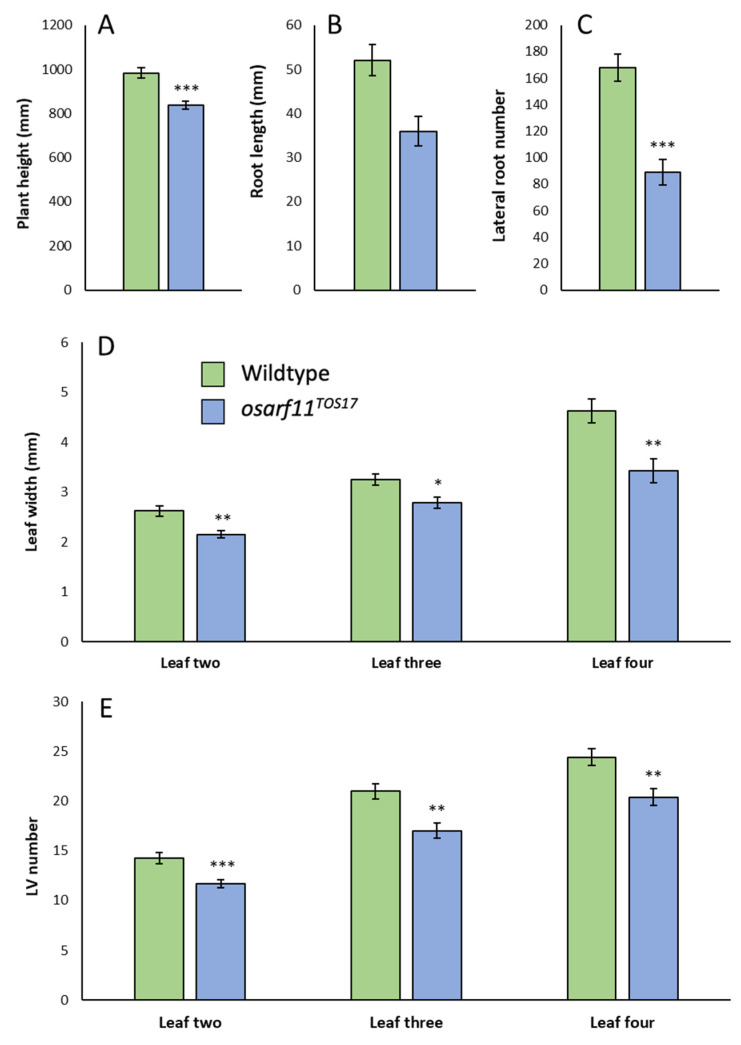
Quantitative phenotypes observed in *osarf11^TOS-17^* plants. (**A**) Plant height of three-month-old adult plants. (**B**) Primary root length and (**C**) lateral root number in three-week-old seedlings. (**D**) Leaf width and (**E**) longitudinal vein, *LV*, number measured in leaves two, three, and four of the primary shoot. Asterisks indicate significant differences with respect to wildtype (Student’s *t* test; * *p* < 0.05, ** *p* < 0.01, *** *p* < 0.001). Error bars = standard error.

To complement the analysis of *osarf11^TRIM^*, we also conducted pairwise comparison of wildtype and homozygous TOS-17 insertion mutant (*osarf11^TOS-17^*). Just as in *osarf11^TRIM^* mutants, we observed that self-fertilized heterozygous *osarf11^TOS-17^* plants segregated dwarf seedlings. As described above, we focused our quantification of the heterogeneous phenotype on homozygous *osarf11^TRIM^* mutant populations. Unlike *osarf11^TRIM^* though, the reduced stature in *osarf11^TOS-17^* mutants remained consistent, manifesting in, on average, 15% shorter shoots in adult plants (Figure 5A). The primary root was 31% shorter in *osarf11^TOS-17^* in three-week old seedlings and the root system had 47% fewer lateral roots (Figure 5B,C) and 12% fewer crown roots (Table S3). We saw a comparable reduction in width of leaves in *osarf11^TRIM^* (17–21%) and *osarf11^TOS-17^* (14–26%) relative to corresponding wildtype backgrounds (Figure 3E, Figure 5D). This defect was companioned by a comparable reduction in vein number in *osarf11^TRIM^* (17–21%) and *osarf11^TOS-17^* (16–19%) (Figure 4A, Figure 5E). 

### 2.5. Loss of OsARF11 Results in Reduced Auxin Perception 

#### 2.5.1. Callus Induction 

**Figure 6:**
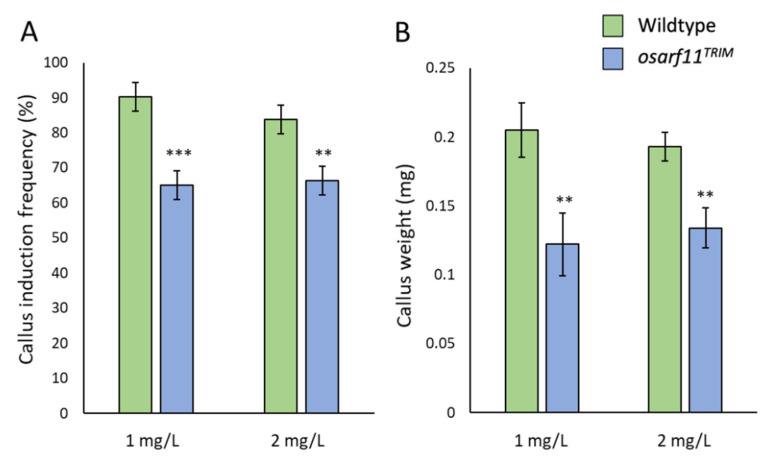
Callus induced from *osarf11^TRIM^* and wildtype seeds with 2,4-Dichlorophenoxyacetic acid (2,4-D). (**A**) Callus induction frequency. Each column represents the average frequency of five trials. Callus induction frequency (%) = number of seeds producing callus/number of seeds inoculated × 100. (**B**) Weight of seed-induced calli after four weeks of 2,4-D exposure. Asterisks indicate significant differences with respect to wildtype (Student’s *t* test; ** *p* < 0.01, *** *p* < 0.001). Error bars = standard error.

Exogenous application of the synthetic auxin 2,4-Dichlorophenoxyacetic acid (2,4-D) results in artificial callus induction in in vitro culture in various plant species. To assess if the *OsARF11* contributes to this auxin response in rice, we evaluated callus induction from germinating seeds. After two weeks on 2,4-D-containing media, germinating *osarf11^TRIM^* seeds had a 28% (1 mg/L 2,4-D) and 21% (2 mg/L 2,4-D) reduced incidence of calli (Figure 6A). We also documented, on average, 41% (1 mg/L 2,4-D) and 31% (2 mg/L 2,4-D) less callus produced from *osarf11^TRIM^* seeds relative to wildtype seeds (Figure 6B). After four weeks of growth, proliferating calli from both genotypes were re-plated on shoot induction media with a high cytokinin concentration. We observed no visual difference in the ability to regenerate shoots between the genotypes. 

#### 2.5.2. IAA Applications

**Figure 7:**
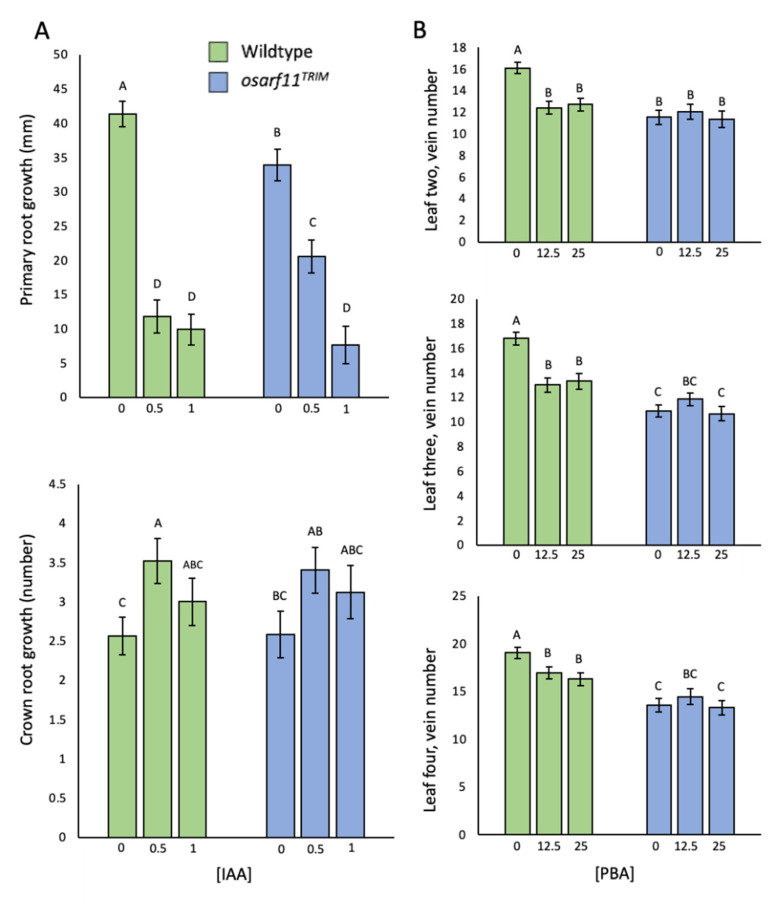
The effect of exogenous auxin and phenylboronic acid (PBA) exposure during early development. (**A**) Shoot and root growth following a seven-day exposure to indole-3-acetic acid (IAA) (0, 0.5, 1 μM) in wildtype and *osarf11^TRIM^* seedlings. (**B**) Longitudinal vein development in the presence of PBA (0, 12.5, 25 μM). Statistical significance was determined using a pair-wise Student’s *t* test. Columns not represented by the same letter, were found to be significantly different. Error bars = standard error.

The effect of exogenous IAA on developing tissues during early root development was evaluated in *osarf11^TRIM^* plants relative to wildtype. Since roots of wildtype and mutant seedlings differ in length and number (Figure 3A–C), we measured the change in growth during the test period rather than resulting phenotypes. After seven days of IAA exposure, the growth of wildtype primary roots was inhibited by 72% (0.5 μM IAA), and 76% (1.0 μM IAA) relative to the control untreated wildtype plants (Figure 7A). The response of mutant seedlings differed in that 0.5 μM IAA inhibited primary root growth by only 40%, and 1.0 μM IAA was required to observe an effect comparable (77%) to 0.5 μM IAA in wildtype roots. Growing the plants in 0.5 μM IAA resulted in a significant increase in the number of crown roots in wildtype seedlings but not in *osarf11^TRIM^* seedlings (Figure 7A). Treatment with 1.0 μM IAA resulted in an insignificant increase in crown root number in both genotypes. 

#### 2.5.3. Phenylboronic Acid Exposure 

In vitro analysis has showed that phenylboronic acid treatment can phenocopy *mp/arf5* phenotypes by inhibition of polar auxin transport [56]. We applied phenylboronic acid (PBA) to germinating wildtype and *osarf11^TRIM^* plants to assess its effect on vein formation. In leaves two, three, and four of wildtype seedlings, exposure to 12.5 and 25 μM PBA resulted in a significant decrease in the number of longitudinal veins (Figure 7B). No such effect was seen in *osarf11^TRIM^* seedlings. 

### 2.6. Gravitropism

**Figure 8:**
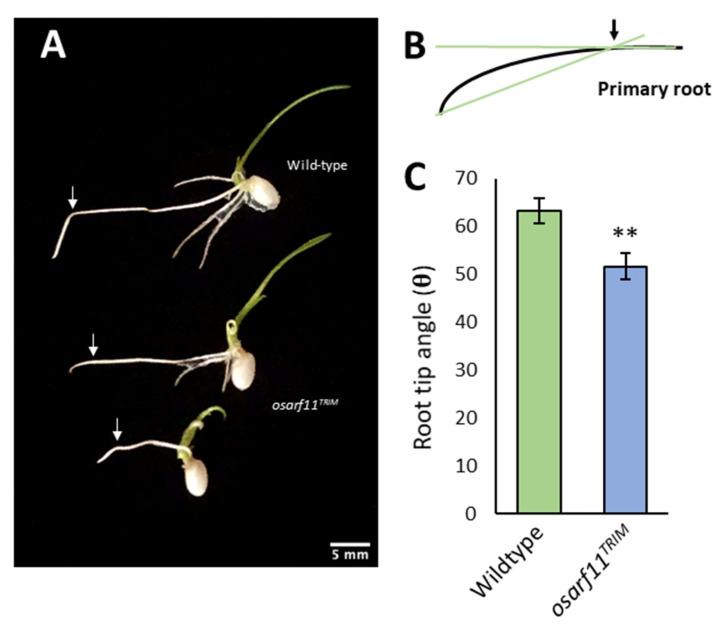
Root gravitropic response in *osarf11^TRIM^* and wildtype seedlings. (**A**) Wildtype and *osarf11^TRIM^* seedlings were grown vertically for six days under normal growth conditions, followed by a 90° rotation. Arrows in (**A**) indicate root tip position at time of rotation, which is used as a base to measure angle of reorientation after 24 h of growth (**B**). Mean root tip angle (**C**). Data from three independent trials were pooled; wildtype, n = 46; *osarf11^TRIM^*, n = 38. Asterisks indicate significant differences with respect to wildtype (Student’s *t* test; ** *p* < 0.01). Error bars = standard error.

As root gravitropism depends on both carrier-mediated asymmetric distribution of auxin in the roots and auxin-mediated cell expansion [57,58], we evaluated the effect of a 90° shift in orientation in *osarf11^TRIM^* and wildtype roots (Figure 8). Root tip angles were variable amongst both genotypes, but on average, wildtype plants showed a root tip angle of 63.24° and *osarf11^TRIM^* a root tip angle of 51.7°, which translates into a 18% reduction in root curvature (*p* = 0.0034) relative to wildtype.

## 3. Discussion 

In this study, we have assessed the phenotypes of plants that are homozygous for insertions in the *OsARF11* gene. The two alleles, generated by independent insertions, resulted in a range of similar phenotypes in homozygous mutants. In addition, many of these traits have been linked to auxin before, including the narrow leaf, which has been described in an *osarf11* mutant, a mutant defective in auxin biosynthesis, and a mutant defective in auxin transport [54,55,59]. Thus, we find it likely that the observed phenotypes are due to the loss of the *OsARF11* function. The two alleles differ in the degree of fertility, though, as *osarf11^TRIM^* produces 45% fewer seeds per panicle than wildtype plants and homozygous *osarf11^TOS-17^* mutants do not produce any seeds. Since the *osarf11^TOS-17^* insertion is in the fifth exon, and the *osarf11^TRIM^* insertion is in the eleventh exon, it is possible that the latter is a weak allele or interferes with gene function differently than the *osarf11^TOS-17^* allele. Both alleles, however, have insertions in the region encoding the amino-terminal DNA-binding domain, truncating both the central activation domain and the carboxy-terminal dimerization domains, indicating that both are null alleles. The TOS-17 line is known to carry seven transposon copies, of which only two are inserted in predicted genes, *OsARF11* and *OsTMF*. Homozygous *ostmf* mutants do not show reduced fertility or other defects characterized here [60], indicating that the increased infertility in the TOS-17 line is not due to a defect in the *OsTMF* gene. An alternative explanation is that *osarf11* effect on fertility is more penetrant in the TOS-17 genome background than in the TRIM background. This explanation seems likely since the mutants differ only quantitatively for this trait. The identification and analysis of additional *osarf11* mutant alleles may be the easiest way of addressing the function of *OsARF11* in fertilization and early embryo development. 

We observed *osarf11* phenotypes that can be summarized as reduced size, reduced number of meristems, fewer veins, fewer seeds, and reduced perception of auxin. Here, we will address these phenotype classes and the role that *OsARF11* may play in corresponding developmental processes. The reduced growth of roots and leaf blade, both in length and width, taken together with the reduced response to exogenous auxin, both in incidence of callus formation and growth of calli, are consistent with a role of *OsARF11* in mediating a response to auxin to stimulate cell division, cell expansion, or both. Likewise, we observed that *osarf11* mutants had reduced response to concentrations of IAA that inhibit root elongation, indicating that *OsARF11* plays a role in auxin-mediated root cell elongation. Auxin is one of the main activators of both cell division and cell expansion [3]. Although evidence for a direct role of ARFs in these processes is sparse [3,61], reduced size of organs is a reoccurring theme in rice *arf* mutants [38,41,43,44,45] and these genes could have both unique and overlapping functions in the regulation of cell division and elongation. The *ds1* rice dwarf mutant is defective in a gene similar to the *A. thaliana EMBRYONIC FLOWER 1 (EMF1)* gene [53]. Yeast-two-hybrid experiments show that DS1 proteins interact with OsARF11 and other proteins and both DS1 and OsARF11 proteins localize to nuclei [53]. This interaction suggests that miss-regulation of *OsARF11* may be part of the *ds1* dwarf phenotype. The *ds1* mutants are also insensitive to brassinosteroids, which in part appears to be mediated by OsARF11, as expression of the brassinosteroid receptor *OsBRI1* is reduced in *osarf11* mutants and OsARF11 can bind to AuxREs in the *OsBRI1* promoter [54] Thus, there is already evidence that OsARF11 influence growth by increasing sensitivity to brassinosteroid growth hormones. 

We observed several instances in which fewer meristem-derived organs formed in the *osarf11* mutants. First, fewer crown and lateral roots formed in *osarf11* seedlings. Second, mutant panicles formed fewer branches, and branches had fewer grains. These phenotypes are consistent with the relatively high level of *OsARF11* expression in developing roots, shoot apical meristem, and young panicles [53]. The reduced panicle branch and grain phenotype is akin to weak alleles of the *A. thaliana MP/ARF5* gene, to which *OsARF11* is the closest rice homolog based on phylogenetic analysis [37]. While strong *mp/arf5* alleles do not form an embryonic root, and after induction of roots, form inflorescences with no flowers, weak alleles form a reduced root system and fewer flowers relative to wildtype plants [22]. *OsARF19* contributes to lateral root formation [62] and *OsARF1* regulates the *CROWN ROOT-LESS 1* gene [63], providing potential partners for *OsARF11* in root meristem development.

Leaves of *osarf11* mutants have a normal number of primary and secondary veins, but a reduced number of tertiary and commissural veins. We suggest several potential causes for this difference. First, the reduced venation is a secondary effect of reduced leaf width. Speaking against this option is the parallel increase in vein spacing in mutant leaves. Second, levels of inductive auxin may be limited at the time of tertiary and commissural vein formation, which combined with reduced auxin perception results in fewer higher-order veins in mutant leaves. However, there are *A. thaliana* mutants with defects in auxin perception, auxin efflux carrier localization, and auxin biosynthesis that have in common more or less extensive gaps in differentiation of late-forming xylem vessels in leaves [22,64,65,66,67]. We see no gaps in the continuity of vein vessels in *osarf11* mutant leaves (data not shown), speaking against this general explanation. Finally, the two groups of veins differ in their origin of appearance. Major monocot veins extend to the leaf margin [68] and may be initiated in a process involving the epidermis similar to that seen in *A. thaliana* [8,26]. In contrast, tertiary and commissural veins form internally, between existing veins, and may depend on a modified vein formation process with a higher dependence on *OsARF11*. We also saw that *osarf11* tertiary veins appear further away from the leaf apex than wildtype tertiary veins (Figure 4E and Figure S5), which may be another facet of reduced response to auxin. The reduced number of veins together with the lower vein density is another *osarf11* phenotype that is similar to *mp/arf5* mutants, albeit not as strong. Although dicot and monocot venation patterns are radically different, the areole area is increased by as much as 37% in *osarf11* leaves (Figure 4H), a number comparable to that seen in a weak *mp/arf5* allele [22]. The large increase in areole area is probably a compound effect of fewer tertiary and commissural veins (Figure 4C,F) and is likely to affect both water supply and removal of photosynthates, which in turn could contribute to other observed reduced-growth phenotypes. Small areole areas have been linked to acclimation and adaptation to drought stress and reduced sugar accumulation feedback on photosynthesis [69,70,71,72], and *OsARF11* could thus play an indirect role in these processes. Although *MP/ARF5* expression precedes and predicts vein formation [25,26], no such information is available for *OsARF11*. Treatment of developing *A. thaliana* embryos with phenylboronic acid (PBA) results in *mp/arf5*-like seedlings including little or no venation in cotyledons, an effect attributed to disruption of polar auxin transport by internalization of PIN1 auxin efflux carriers [56]. In rice seedlings germinating on PBA-containing media, the effect was much less dramatic, causing reduced numbers of veins in leaves of wildtype seedlings but not *osarf11^TRIM^* seedlings. In strict genetic terms, the insensitivity suggests that *OsARF11* is epistatic to the function inhibited by PBA, possibly a rice *PIN1* ortholog, indicating that a function of OsARF11 could be to activate the expression of an *OsPIN* gene, similar to the MP/ARF5 regulation of *PIN1* in *A. thaliana* [26,73]. Similar to MP/ARF5, there is also evidence that steady-state levels of *OsARF11* transcripts increase in response to IAA treatment [53,74], which would allow *OsARF11* to respond also on a transcriptional level to local auxin maxima formed during vein and meristem specification.

The reduced fecundity as well as the range of early seedling phenotypes in *osarf11* mutants are enigmatic. It is possible that they are all part of a spectrum of stochastic phenotypes caused by auxin signaling instability in the absence of *OsARF11*. Mutants in the *GNOM* gene, defective in endosomal recycling needed for polar auxin transport, provide precedent for a situation in which a single gene disruption can result in a wide range of embryo and seedling phenotypes [75,76]. A cursory assessment of *osarf11* embryo anatomy, however, did not reveal obvious defects (not shown) and gene expression markers of auxin-related processes and tissues will be needed to understand the role of *OsARF11* in embryo development and early seedling growth. 

Our analyses provide evidence that *OsARF11* contributes substantially to seedling growth, leaf, and root system development. We also saw a 45% reduction in seed number per plant and a 17% reduction in seed weight in *osarf11* mutants. Differently from the *osarf11* alleles in this study, varieties used for breeding are likely to carry functional allele variants of the *OsARF11* that differ considerably less in their contribution to important quantitative traits. The fact remains, however, that this study pinpoints a role of *OsARF11* in these processes, providing a candidate for selection and targeted modification of rice productivity. 

## 4. Materials and Methods

### 4.1. Gene Terminology, Mutant Lines, Genotyping, and Growth Conditions

Upper case indicates wildtype allele, lower case indicates mutant allele. Italic font indicates gene or transcript, and regular font indicates protein. Sequence and annotation for *OsARF11* can be found at the Rice Annotation Project Database [77] using accession no. Os04g0664400, National Center for Biotechnology Information using accession no. LOC4337309 or Michigan State University accession no. LOC_Os04g56850.1. This gene is also known as *OsARF11-like, OsARF5*, and *OsMP*. Mutant lines containing insertional mutations in the *OsARF11* predicted open-reading frame were kindly provided by both TRIM (M0030446, *OsARF6*) [49] and TOS-17 (NC2659) [50] databases. The sequence information available at the mutant line websites indicate one T-DNA insertion in the M0030446 line and two gene TOS-17 insertions in the NC2659 line, with an overlap only in the *OsARF11* gene. The *Oryza sativa japonica* variety Nipponbare, in which the mutations were introduced, was used as a wildtype control in all comparison studies. DNA was extracted from 14-day-old seedlings using ChargeSwitch gDNA Plant Kit (CS18000, ThermoFisher Scientific, Burlington, ON, Canada). PCR was used to confirm the insertion and identify homozygous mutant plants in segregating populations (Supplemental Figure S1). T-DNA primer 5′-TCGCGATCCAGACTGAATGC-3′ and *OsARF11* primer 5′-GGGGACTCCCAAGGGTTTGA-3′ were used to confirm the insertion of the T-DNA in *osarf11^TRIM^*. *OsARF11^Tos-17^* primer 5′-CAGAAATATTCAGTGGGGTG-3′ and tail6 TOS-17 primer 5′-AGGTTGCAAGTTAGTTAAGA-3′ were used to confirm the *TOS-17* tag in *osarf11^TOS-17^*. 

Morphometric measurements were scored in wildtype, *osarf11^TRIM^*, and *osarf11^TOS-17^* lines. Full seeds were de-husked, and surface sterilized in 70% (*v*/*v*) ethanol for 5 min, followed by 50% (*v*/*v*) commercial bleach with continuous shaking for 30 min and repeated washes with sterile water. Seeds were dried on sterile filter paper and plated on half-strength Murashige and Skoog Medium (MS, M5531, PhytoTech Labs Inc., Lenexa KS, USA) supplemented with 30 g/L sucrose and 8 g/L plant agar (A111, PhytoTech Labs Inc., Lenexa, KS, USA). Plates were incubated in a growth chamber for seven days at 25 °C with 16/8-h light/dark conditions. Germinated seedlings were transferred to beakers with distilled water containing 0.2 g/L NPK 20-20-20 fertilizer for one week followed by potting in clay particle media (Turface MVP, Evergro, Delta, BC, Canada) as described (Eddy et al., 2008.) and moved to a greenhouse. Natural light was supplemented with 600 W high-pressure sodium lamps up to 12 h/day and temperature was kept between 20 and 30 °C. Plants were fertilized twice a week, alternating between 2 g/L NPK 20-20-20 and 0.2 g/L NPK 21-0-0. Mutant seedlings displaying extreme dwarfism and insufficient root development did not survive the transfer to clay media and were not included in adult phenotype scoring. 

### 4.2. Tissue Culture Assays

Callus tissue was induced from wildtype and *osarf11^TRIM^* de-husked seeds plated on full strength MS media supplemented with 30 g/L sucrose, 30 mg/L casamino acids, 2.8 g/L L-proline, 4 g/L phytagel (P8169, Sigma-Aldrich Canada Co., Oakville, ON, Canada) and 1 or 2 mg/L 2,4-Dichlorophenoxyacetic acid (2,4-D). Media was buffered to a pH of 5.8. Plates were placed in an incubator set to 30 °C with approximately 200 µmol m^−2^ s^−1^ light intensity and 16-h day length. The frequency of seeds that produced callus were scored after two weeks. Proliferating calli were transferred to fresh media and grown for an additional two weeks, after which their weights were taken. To induce shoots, calli were transferred from media described above, but supplemented with 2 mg/L Kinetin and 1 mg/L NAA, instead of 2,4-D. Cultures were transferred to a growth chamber set to 25 °C with 16/8-h light/dark conditions.

Seven-day-old wildtype and *osarf11^TRIM^* seedlings were exposed to 0, 0.5, or 1 μM Indole Acetic Acid (IAA) while in liquid culture (0.2 g/L NPK 20-20-20). Solutions containing 0 μM IAA were inoculated with a mock dose containing DMSO, which was used as a hormone solvent. Root and shoot growth were measured prior to and following seven days of auxin exposure. The difference in growth during the treatment period = Trait X post-treatment minus Trait X pre-treatment. In a separate experiment, wildtype and *osarf11^TRIM^* seeds were germinated on half-strength MS supplemented with 0, 12.5, and 25 μM phenylboronic acid (PBA, BB-2375, Combi-Blocks, Inc., San Diego, CA, USA) to determine the effect of PBA on longitudinal vein development in the second, third, and fourth leaves of the primary shoot.

For root gravitropism responses, seeds from both genotypes were first plated on half strength MS and grown vertically for six days. Plates were thereafter rotated at a 90-degree angle and evaluated after 24 h. Root curvature was measured using ImageJ software [78] as described [63].

### 4.3. Microscopy

Seedlings were fixed overnight in a 6:1 solution of ethanol to acetic acid, followed by a brief wash in 50% ethanol. Individual leaves were collected and cleared in lactic acid by heating to 95 °C for 30 min. Explants were mounted in a 30% aqueous glycerol solution. Leaf venation was observed using differential interference contrast in a Nikon Eclipse E600 microscope (EquipNet, Inc. Canton, MA, USA). Images were captured with a Canon EOS 5D Mark II camera (Henry’s Camera, Vancouver, BC, Canada).

### 4.4. Statistical Analysis

Statistical analyses were conducted in JMP (SAS, Cary, NC, USA) using a one-way analysis of variance (ANOVA) test and Student’s *t* test. Associated graphs were generated in Excel. As *osarf11* mutant phenotypes were not fully penetrant, measurements from repeat trials were combined into a single data set to increase sample size. Two or more growth trials were conducted per experiment.

## 5. Conclusions

The *OsARF11* gene has appeared in gene expression studies and its protein as an interactor with other proteins with key roles in rice development, but a thorough analysis of its function based on mutant analysis has to-date been lacking. This study fills that gap in knowledge and provides evidence that *OsARF11* acts in auxin-dependent processes throughout the rice life cycle. *OsARF11* promotes both root and leaf growth, formation of roots, panicle branches, grains, and higher-order veins. We also found evidence for a role in auxin perception, response to gravitropic stimulus, as well as seed formation and development. While *osarf11* seedlings did not display the patterning defects seen in mutants of its potential *A. thaliana* ortholog *MP/ARF5*, we saw a partial overlap in vein and reproductive lateral organ formation, suggesting that *OsARF11* may have functions similar to *MP/ARF5* in some processes. This study provides the baseline needed for more detailed studies on the role of *OsARF11* in these developmental processes and suggests a role of *OsARF11* in key quantitative traits in this economically important species. 

## Supplementary Materials

The following are available online at https://www.mdpi.com/article/10.3390/ijms22084089/s1, Figure S1: Identification of homozygous *Osarf11^TOS1^*^7^ mutants. Figure S2: Sequence of TRIM T-DNA 3′ end insertion point. Figure S3: Insertion of TOS17 transposon in the *OsARF11* gene. Figure S4: Height phenotype of two-month-old *osarf11* mutants. Figure S5: Tertiary vein initiation in *osarf11^TRIM^* relative to wildtype. Table S1: Quantitative phenotypes measured in *osarf11^TRIM^* plants. Table S2: Leaf venation defects in *osarf11^TRIM^* seedlings., Table S3: Quantitative phenotypes observed in *osarf11^TOS−17^* plants.

## References

[1] Kajala K., Covshoff S., Karki S., Woodfield H., Tolley B.J., Dionora M.J.A., Mogul R.T., Mabilangan A.E., Danila F.R., Hibberd J.M. (2011). Strategies for engineering a two-celled C 4 photosynthetic pathway into rice. J. Exp. Bot..

[2] Wang P., Vlad D., Langdale J.A. (2016). Finding the genes to build C4 rice. Curr. Opin. Plant Biol..

[3] Perrot-Rechenmann C. (2010). Cellular responses to auxin: Division versus expansion. Cold Spring Harb. Perspect. Biol..

[4] Scarpella E., Helariutta Y. (2010). Vascular Pattern Formation in Plants. Curr. Top. Dev. Biol..

[5] Aloni R. (2010). The Induction of Vascular Tissues by Auxin. Plant Hormones.

[6] Smit M.E., Weijers D. (2015). The role of auxin signaling in early embryo pattern formation. Curr. Opin. Plant Biol..

[7] Reinhardt D., Pesce E.R., Stieger P., Mandel T., Baltensperger K., Bennett M., Traas J., Friml J., Kuhlemeier C. (2003). Regulation of phyllotaxis by polar auxin transport. Nature.

[8] Scarpella E., Marcos D., Friml J., Berleth T. (2006). Control of leaf vascular patterning by polar auxin transport. Genes Dev..

[9] Weijers D., Wagner D. (2016). Transcriptional Responses to the Auxin Hormone. Annu. Rev. Plant Biol..

[10] Chandler J.W. (2016). Auxin response factors. Plant Cell Environ..

[11] Vernoux T., Brunoud G., Farcot E., Morin V., Van Den Daele H., Legrand J., Oliva M., Das P., Larrieu A., Wells D. (2011). The auxin signalling network translates dynamic input into robust patterning at the shoot apex. Mol. Syst. Biol..

[12] Calderón Villalobos L.I.A., Lee S., De Oliveira C., Ivetac A., Brandt W., Armitage L., Sheard L.B., Tan X., Parry G., Mao H. (2012). A combinatorial TIR1/AFB–Aux/IAA co-receptor system for differential sensing of auxin. Nat. Chem. Biol..

[13] Powers S.K., Strader L.C. (2020). Regulation of auxin transcriptional responses. Dev. Dyn..

[14] Kepinski S., Leyser O. (2005). The *Arabidopsis* F-box protein TIR1 is an auxin receptor. Nature.

[15] Sharon M., Zheng C., Zheng N., Calderon-Villalobos L.I.A., Estelle M., Tan X., Robinson C.V. (2007). Mechanism of auxin perception by the TIR1 ubiquitin ligase. Nature.

[16] Ulmasov T. (1997). ARF1, a Transcription Factor That Binds to Auxin Response Elements. Science.

[17] Freire-Rios A., Tanaka K., Crespo I., Van der Wijk E., Sizentsova Y., Levitsky V., Lindhoud S., Fontana M., Hohlbein J., Roeland Boer D. (2020). Architecture of DNA elements mediating ARF transcription factor binding and auxin-responsive gene expression in Arabidopsis. Proc. Natl. Acad. Sci. USA.

[18] Pierre-Jerome E., Moss B.L., Lanctot A., Hageman A., Nemhauser J.L. (2016). Functional analysis of molecular interactions in synthetic auxin response circuits. Proc. Natl. Acad. Sci. USA.

[19] Stigliani A., Martin-Arevalillo R., Lucas J., Bessy A., Vinos-Poyo T., Mironova V., Vernoux T., Dumas R., Parcy F. (2019). Capturing auxin response factors syntax using DNA binding models. Mol. Plant.

[20] Wu M.F., Yamaguchi N., Xiao J., Bargmann B., Estelle M., Sang Y., Wagner D. (2015). Auxin-regulated chromatin switch directs acquisition of flower primordium founder fate. Elife.

[21] Roosjen M., Paque S., Weijers D. (2018). Auxin Response Factors: Output control in auxin biology. J. Exp. Bot..

[22] Przemeck G.K.H., Mattsson J., Hardtke C.S.C.S., Sung Z.R.R., Berleth T. (1996). Studies on the role of the *Arabidopsis* gene MONOPTEROS in vascular development and plant cell axialization. Planta.

[23] Berleth T., Jurgens G. (2008). The role of the monopteros gene in organising the basal body region of the *Arabidopsis* embryos. Trends Genet..

[24] Hardtke C.S., Ckurshumova W., Vidaurre D.P., Singh S.A., Stamatiou G., Tiwari S.B., Hagen G., Guilfoyle T.J., Berleth T. (2004). Overlapping and non-redundant functions of the *Arabidopsis* auxin response factors MONOPTEROS and NONPHOTOTROPIC HYPOCOTYL 4. Development.

[25] Hardtke C.S., Berleth T. (1998). The *Arabidopsis* gene MONOPTEROS encodes a transription factor mediating embryo axis formation and vascular development. EMBO J..

[26] Wenzel C.L., Schuetz M., Yu Q., Mattsson J. (2007). Dynamics of MONOPTEROS and PIN-FORMED1 expression during leaf vein pattern formation in *Arabidopsis thaliana*. Plant J..

[27] Schuetz M., Berleth T., Mattsson J. (2008). Multiple MONOPTEROS-dependent pathways are involved in leaf initiation. Plant Physiol..

[28] Schlereth A., Möller B., Liu W., Kientz M., Flipse J., Rademacher E.H., Schmid M., Jürgens G., Weijers D. (2010). MONOPTEROS controls embryonic root initiation by regulating a mobile transcription factor. Nature.

[29] Bhatia N., Bozorg B., Larsson A., Ohno C., Jönsson H., Heisler M.G. (2016). Auxin Acts through MONOPTEROS to Regulate Plant Cell Polarity and Pattern Phyllotaxis. Curr. Biol..

[30] Nemhauser J.L., Feldman L.J., Zambryski P.C. (2000). Auxin and ETTIN in *Arabidopsis* gynoecium morphogenesis. Development.

[31] Simonini S., Deb J., Moubayidin L., Stephenson P., Valluru M., Freire-Rios A., Sorefan K., Weijers D., Friml J., Østergaard L. (2016). A noncanonical auxin-sensing mechanism is required for organ morphogenesis in arabidopsis. Genes Dev..

[32] Ellis C.M., Nagpal P., Young J.C., Hagen G., Guilfoyle T.J., Reed J.W. (2005). AUXIN RESPONSE FACTOR1 and AUXIN RESPONSE FACTOR2 regulate senescence and floral organ abscission in Arabidopsisthaliana. Development.

[33] Shin R., Burch A.Y., Huppert K.A., Tiwari S.B., Murphy A.S., Guilfoyle T.J., Schachtman D.P. (2007). The *Arabidopsis* Transcription Factor MYB77 Modulates Auxin Signal Transduction. Plant Cell Online.

[34] Salehin M., Li B., Tang M., Katz E., Song L., Ecker J.R., Kliebenstein D.J., Estelle M. (2019). Auxin-sensitive Aux/IAA proteins mediate drought tolerance in *Arabidopsis* by regulating glucosinolate levels. Nat. Commun..

[35] Perez-Torres C.-A., Lopez-Bucio J., Cruz-Ramirez A., Ibarra-Laclette E., Dharmasiri S., Estelle M., Herrera-Estrella L. (2008). Phosphate Availability Alters Lateral Root Development in *Arabidopsis* by Modulating Auxin Sensitivity via a Mechanism Involving the TIR1 Auxin Receptor. Plant Cell.

[36] Sato Y., Nishimura A., Ito M., Ashikari M., Hirano H.-Y., Matsuoka M. (2001). Auxin response factor family in rice. Genes Genet. Syst..

[37] Wang D., Pei K., Fu Y., Sun Z., Li S., Liu H., Tang K., Han B., Tao Y. (2007). Genome-wide analysis of the auxin response factors (ARF) gene family in rice (*Oryza sativa*). Gene.

[38] Attia K.A., Abdelkhalik A.F., Ammar M.H., Wei C., Yang J., Lightfoot D.A., El-Sayed W.M., El-Shemy H.A. (2009). Antisense phenotypes reveal a functional expression of OsARF1, an auxin response factor, in transgenic rice. Curr. Issues Mol. Biol..

[39] Shen C., Yue R., Sun T., Zhang L., Yang Y., Wang H. (2015). OsARF16, a transcription factor regulating auxin redistribution, is required for iron deficiency response in rice (*Oryza sativa* L.). Plant Sci..

[40] Shen C., Yue R., Yang Y., Zhang L., Sun T., Tie S., Wang H. (2014). OsARF16 is involved in cytokinin-mediated inhibition of phosphate transport and phosphate signaling in rice (*Oryza sativa* L.). PLoS ONE.

[41] Wang S., Zhang S., Sun C., Xu Y., Chen Y., Yu C., Qian Q., Jiang D.A., Qi Y. (2014). Auxin response factor (OsARF12), a novel regulator for phosphate homeostasis in rice (*Oryza sativa*). New Phytol..

[42] Qi Y., Wang S., Shen C., Zhang S., Chen Y., Xu Y., Liu Y., Wu Y., Jiang D. (2012). OsARF12, a transcription activator on auxin response gene, regulates root elongation and affects iron accumulation in rice (*Oryza sativa*). New Phytol..

[43] Huang J., Li Z., Zhao D. (2016). Deregulation of the OsmiR160 target gene OsARF18 causes growth and developmental defects with an alteration of auxin signaling in rice. Sci. Rep..

[44] Sakamoto T., Inukai Y. (2013). Characterization of a *Tos*17 Insertion Mutant of Rice Auxin Signal Transcription Factor Gene, *OsARF*24. Am. J. Plant Sci..

[45] Zhang S., Wu T., Liu S., Liu X., Jiang L., Wan J. (2016). Disruption of OsARF19 is Critical for Floral Organ Development and Plant Architecture in Rice (*Oryza sativa* L.). Plant Mol. Biol. Report..

[46] Zhang S., Wang S., Xu Y., Yu C., Shen C., Qian Q., Geisler M., Jiang D.A., Qi Y. (2015). The auxin response factor, OsARF19, controls rice leaf angles through positively regulating OsGH3-5 and OsBRI1. Plant Cell Environ..

[47] Chen S.H., Zhou L.J., Xu P., Xue H.W. (2018). SPOC domain-containing protein Leaf inclination3 interacts with LIP1 to regulate rice leaf inclination through auxin signaling. PLoS Genet..

[48] Li Y., Li J., Chen Z., Wei Y., Qi Y., Wu C. (2020). OsmiR167a-targeted auxin response factors modulate tiller angle via fine-tuning auxin distribution in rice. Plant Biotechnol. J..

[49] Hsing Y.I., Chern C.G., Fan M.J., Lu P.C., Chen K.T., Lo S.F., Sun P.K., Ho S.L., Lee K.W., Wang Y.C. (2007). A rice gene activation/knockout mutant resource for high throughput functional genomics. Plant Mol. Biol..

[50] Hirochika H. (2001). Contribution of the Tos17 retrotransposon to rice functional genomics. Curr. Opin. Plant Biol..

[51] Song Y., Wang L., Xiong L. (2009). Comprehensive expression profiling analysis of OsIAA gene family in developmental processes and in response to phytohormone and stress treatments. Planta.

[52] Waese J., Fan J., Pasha A., Yu H., Fucile G., Shi R., Cumming M., Kelley L.A., Sternberg M.J., Krishnakumar V. (2017). ePlant: Visualizing and exploring multiple levels of data for hypothesis generation in plant biology. Plant Cell.

[53] Liu X., Yang C.Y., Miao R., Zhou C.L., Cao P.H., Lan J., Zhu X.J., Mou C.L., Huang Y.S., Liu S.J. (2018). DS1/OsEMF1 interacts with OsARF11 to control rice architecture by regulation of brassinosteroid signaling. Rice.

[54] Sakamoto T., Morinaka Y., Inukai Y., Kitano H., Fujioka S. (2013). Auxin signal transcription factor regulates expression of the brassinosteroid receptor gene in rice. Plant J..

[55] Qi J., Qian Q., Bu Q., Li S., Chen Q., Sun J., Liang W., Zhou Y., Chu C., Li X. (2008). Mutation of the rice narrow leaf1 gene, which encodes a novel protein, affects vein patterning and polar auxin transport. Plant Physiol..

[56] Matthes M., Torres-Ruiz R.A. (2016). Boronic acid treatment phenocopies monopteros by affecting PIN1 membrane stability and polar auxin transport in *Arabidopsis thaliana* embryos. Development.

[57] Band L.R., Wells D.M., Larrieu A., Sun J., Middleton A.M., French A.P., Brunoud G., Sato E.M., Wilson M.H., Peŕet B. (2012). Root gravitropism is regulated by a transient lateral auxin gradient controlled by a tipping-point mechanism. Proc. Natl. Acad. Sci. USA.

[58] Ottenschläger I., Wolff P., Wolverton C., Bhalerao R.P., Sandberg G., Ishikawa H., Evans M., Palme K. (2003). Gravity-regulated differential auxin transport from columella to lateral root cap cells. Proc. Natl. Acad. Sci. USA.

[59] Fujino K., Matsuda Y., Ozawa K., Nishimura T., Koshiba T., Fraaije M.W., Sekiguchi H. (2008). NARROW LEAF 7 controls leaf shape mediated by auxin in rice. Mol. Genet. Genom..

[60] Xu Y., Hu D., Hou X., Shen J., Liu J., Cen X., Fu J., Li X., Hu H., Xiong L. (2020). OsTMF attenuates cold tolerance by affecting cell wall properties in rice. New Phytol..

[61] Du M., Spalding E.P., Gray W.M. (2020). Rapid Auxin-Mediated Cell Expansion. Annu. Rev. Plant Biol..

[62] Wang R., Zhang Y., Kieffer M., Yu H., Kepinski S., Estelle M. (2016). HSP90 regulates temperature-dependent seedling growth in *Arabidopsis* by stabilizing the auxin co-receptor F-box protein TIR1. Nat. Commun..

[63] Inukai Y., Sakamoto T., Ueguchi-Tanaka M., Shibata Y., Gomi K., Umemura I., Hasegawa Y., Ashikari M., Kitano H., Matsuoka M. (2005). Crown rootless1, which is essential for crown root formation in rice, is a target of an Auxin Response Factor in auxin signaling. Plant Cell.

[64] Mariyamma N.P., Hou H., Carland F.M., Nelson T., Schultz E.A. (2017). Localization of *Arabidopsis* FORKED1 to a RABA-positive compartment suggests a role in secretion. J. Exp. Bot..

[65] Prabhakaran Mariyamma N., Clarke K.J., Yu H., Wilton E.E., Van Dyk J., Hou H., Schultz E.A. (2018). Members of the *Arabidopsis* FORKED1-LIKE gene family act to localize PIN1 in developing veins. J. Exp. Bot..

[66] Sieburth L.E., Muday G.K., King E.J., Benton G., Kim S., Metcalf K.E., Meyers L., Seamen E., Van Norman J.M. (2006). SCARFACE encodes an ARF-GAP that is required for normal auxin efflux and vein patterning in *Arabidopsis*. Plant Cell.

[67] Baylis T., Cierlik I., Sundberg E., Mattsson J. (2013). SHORT INTERNODES/STYLISH genes, regulators of auxin biosynthesis, are involved in leaf vein development in *Arabidopsis thaliana*. New Phytol..

[68] Nelson T., Dengler N. (1997). Leaf vascular pattern formation. Plant Cell.

[69] Pantin F., Simonneau T., Muller B. (2012). Coming of leaf age: Control of growth by hydraulics and metabolics during leaf ontogeny. New Phytol..

[70] Feldman A.B., Leung H., Baraoidan M., Elmido-Mabilangan A., Canicosa I., Quick W.P., Sheehy J., Murchie E.H. (2017). Increasing leaf vein density via mutagenesis in rice results in an enhanced rate of photosynthesis, smaller cell sizes and can reduce interveinal mesophyll cell number. Front. Plant Sci..

[71] Nardini A., Õunapuu-Pikas E., Savi T. (2014). When smaller is better: Leaf hydraulic conductance and drought vulnerability correlate to leaf size and venation density across four *Coffea arabica* genotypes. Funct. Plant Biol..

[72] Scoffoni C., Chatelet D.S., Pasquet-Kok J., Rawls M., Donoghue M.J., Edwards E.J., Sack L. (2016). Hydraulic basis for the evolution of photosynthetic productivity. Nat. Plants.

[73] Mattsson J., Ckurshumova W., Berleth T. (2003). Auxin Signaling in *Arabidopsis* Leaf Vascular Development. Plant Physiol..

[74] Wenzel C.L., Hester Q., Mattsson J. (2008). Identification of genes expressed in vascular tissues using NPA-induced vascular overgrowth in *Arabidopsis*. Plant Cell Physiol..

[75] Geldner N., Anders N., Wolters H., Keicher J., Kornberger W., Muller P., Delbarre A., Ueda T., Nakano A., Jürgens G. (2003). The *Arabidopsis* GNOM ARF-GEF mediates endosomal recycling, auxin transport, and auxin-dependent plant growth. Cell.

[76] Mayer U., Ruiz R.A.T., Berleth T., Miseéra S., Jürgens G. (1991). Mutations affecting body organization in the *Arabidopsis* embryo. Nature.

[77] Sakai H., Lee S.S., Tanaka T., Numa H., Kim J., Kawahara Y., Wakimoto H., Yang C.C., Iwamoto M., Abe T. (2013). Rice annotation project database (RAP-DB): An integrative and interactive database for rice genomics. Plant Cell Physiol..

[78] Schneider C.A., Rasband W.S., Eliceiri K.W. (2012). NIH Image to ImageJ: 25 years of image analysis. Nat. Methods.

