# Supplementary material for: OsARF11 Promotes Growth, Meristem, Seed, and Vein Formation during Rice Plant Development
Source: Int J Mol Sci. 2021 Apr 15;22(8):4089. doi: 10.3390/ijms22084089 (PMC8071273; doi:10.3390/ijms22084089)
Supplement: Supplementary file 1 [file ijms-22-04089-s001.zip › ijms-1158192-supplementary.pdf]

OsARF11 promotes growth, meristem, seed and vein formation during rice plant development

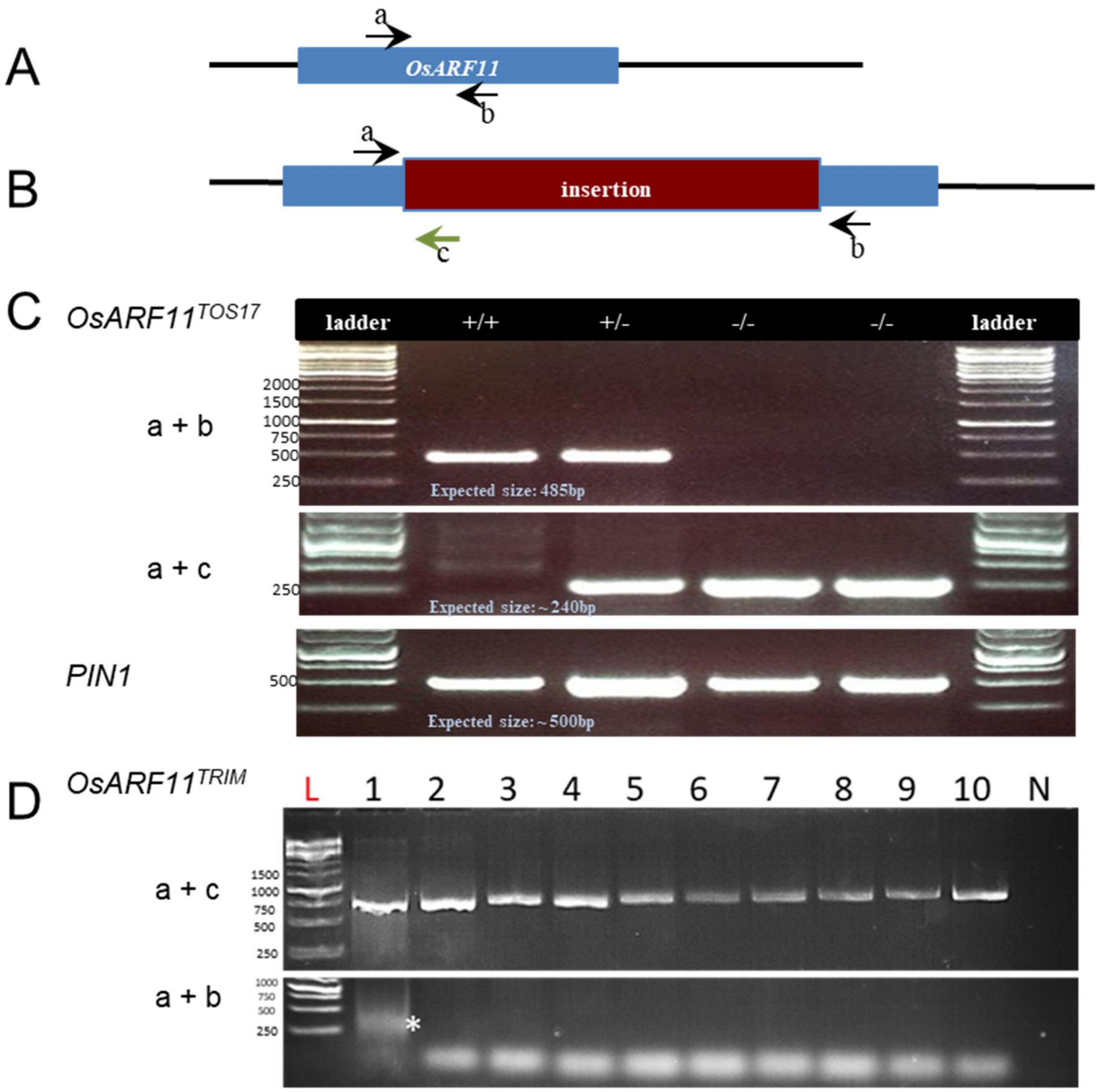

Figure S1. Identification of homozygous *Osarf11*^{TOS17} mutants. DNA purified from seedlings were used for PCR to identify (A) wildtype alleles using primers a+b, (B) mutant insertion using primer a in combination with insert-specific primer c. Primer-binding sites a and b are too far apart to allow amplification under tested conditions. (C) PCR from osarf11^{TOS17} mutants; +/+ denotes plant that tested homozygous for wildtype allele based on presence of PCR product only from PCR using primers a and b; +/- denotes plant DNA sample from which both wildtype (a+b) and mutant (a+c) alleles could be amplified indicating hemizygous plant; -/- denotes plant testing homozygous for insertion based on amplification with primers a+c only. *OsPIN1* gene was used as a positive control for DNA and reagent denotes plant DNA sample from which only mutant allele (D) Amplification of *Osarf11*^{TRIM} insertion allele in DNA from plants 1-10 using primers a+c; DNA from plant 1 tested positive (see asterisk) using a+b primer amplification indicating hemizygous mutant, DNA from plants 2-9 tested negative using a+b primer, indicating that these plants are homozygous mutants. Primers can be found in materials and methods.

Supplemental figures, Sims et. al., 2021

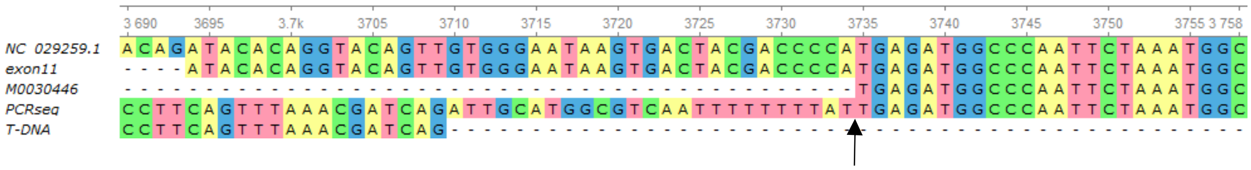

Figure S2. Sequence of TRIM T-DNA 3′ end insertion point. Genomic sequence of *OsARF11* (NC_029259.1), exon 11 in which the right border of T-DNA is inserted, TRIM database insertion sequence (M0030446), sequence of amplicon from above PCR genotyping, end of sequence matching transfer DNA of binary plasmids (T-DNA). Insertion point at this end (arrow).

```
tail6 TOS-17 primer: AGGTTGCAAGTTAGTTAAGA
OsARF11^Tos-17 primer: CAGAAATATTCAGTGGGGTG

PRODUCT SIZE: 397

  1  TGTTAAATATATATACAAGCTAATGTACTGTATAGTTGGCCCATGTCCAGCCCATCGGAT

 61  GTCCAGTCCATTGGATCTTGTATCTTGTATATACTTCTCTATTGCTAATACTATTGTTAG
                                                               >>

121  GTTGCAAGTTAGTTAAGATGGTATCAGAGCAATGGTCTGAACCCTAGCTGCCAATTCCCC
     >>>>>>>>>>>>>>>>>>

181  ATCGCCGGCCGGCGCGGCGGCGTGCCGCCGGCGTTTCACTGCTAAGCAACCTCCTCCAGA

241  CCCTTCAGCCAGTAAACTCTGTAAGTATGATGTTTCTCTGTTACATTGTGTACTGAGTTC

                                                       AATGCATAAA
301  TGTACTACTTTATTTGGTTCTATTAAAATGTAAACACCAATTGTTATTGAAATGCATAAA

     ATGTGATTACAGAAACAAAAATTCAGTGTGCAAGGAATAAAAGATGGATTCTAAATTAAT
361  ATGTGATTACAGAAACAAAAATTCAGTGTGCAAGGAATAAAAGATGGATTCTAAATTAAT

     ATTTATTTGTGCTGTCTATTGTAGGAAACGGATGTATTCCCTATTCCAACTCTTGGTGCT
421  ATTTATTTGTGCCGTCTATTGTAGGAAACGGATGTATTCCCTATTCCAACTCTTGGTGCT

     TATACCAAAAGCAAGCACCCCACTGAATATTTCTG
481  TATACCAAAAGCAAGCACCCCACTGAATATTTCTGCAAGAATTTAACCGCAAGTGATACG
                     <<<<<<<<<<<<<<<<<<<<

541  AGTACACATGGTGGTTTCTCGGTGCCACGAAGGGCTGCAGAGAAGTTGTTCCCACAANTG

601  GTATAGCCTTTATCTACATTTTGTTTGCCGCATAGTTTTTCTGTAAAAAAATGCTTCAGC
```

Figure S3. Insertion site of Tos17 transposon in OsARF11 gene. Sequence in yellow indicates Tos17 transposon (Hirochika, et. al., 1996, Retrotransposons of rice involved in mutations induced by tissue culture. *PNAS*, *93*:7783-7788). Red sequence indicates accession NC2659_0_403_1A, 3' flanking sequence of Tos17 insertion in rice strain NC2659. Green indicates reliable sequence from sequencing of amplicon obtained using indicated primers.

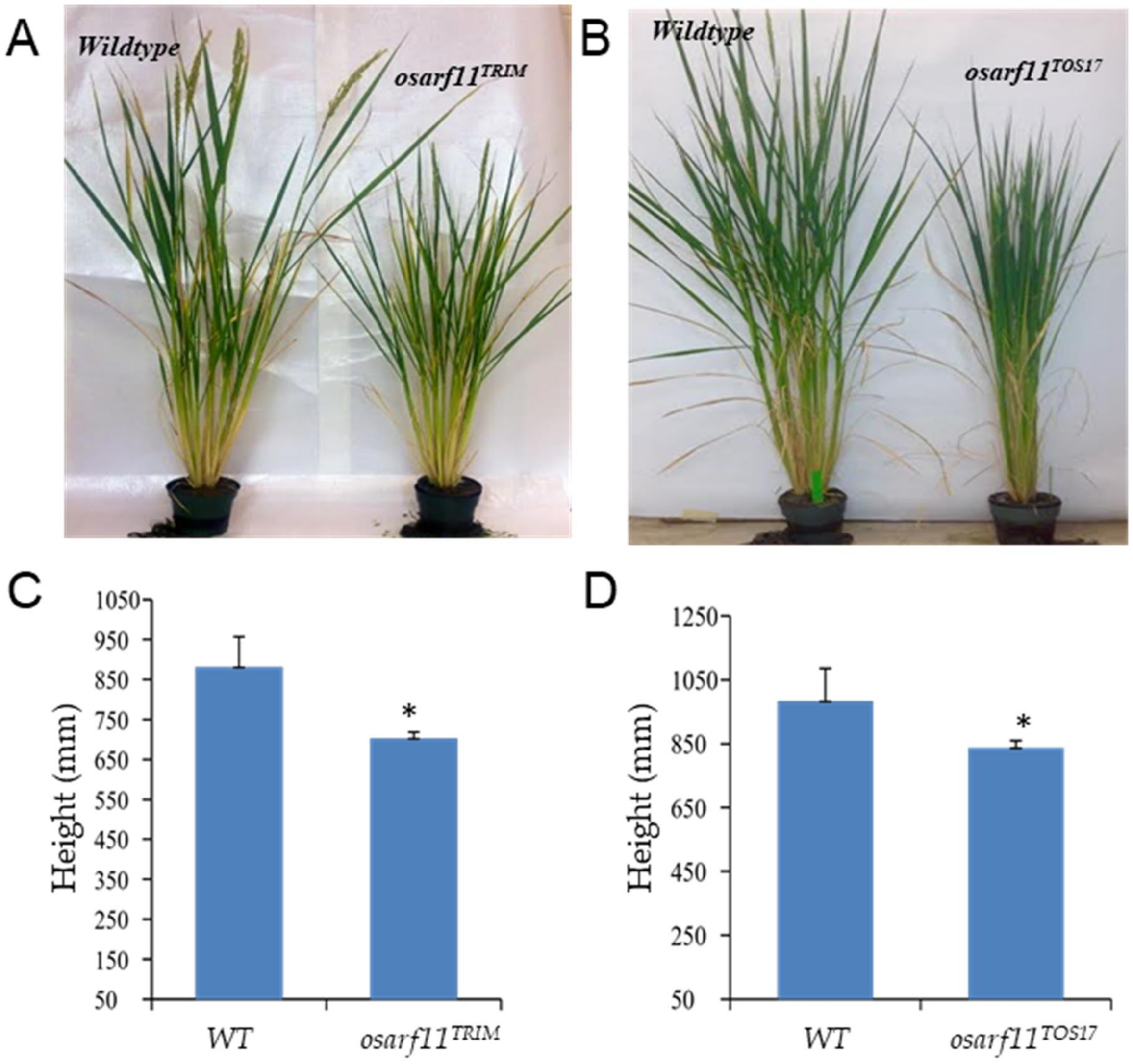

Figure S4. Height phenotype of two-month-old *osarf11* mutants. (A) wildtype of and *osarf11*^{TRIM} mutant; (B) wildtype of and *osarf11*^{TOS17} mutant. The mean height of 6 plants for each genotype: (C) wildtype versus *osarf11*^{TRIM}; wildtype versus *osarf11*^{TOS17}. Height in mm. Asterisks indicate that differences in average height between wild type and mutant plants are significant at a confidence level of > 99%.

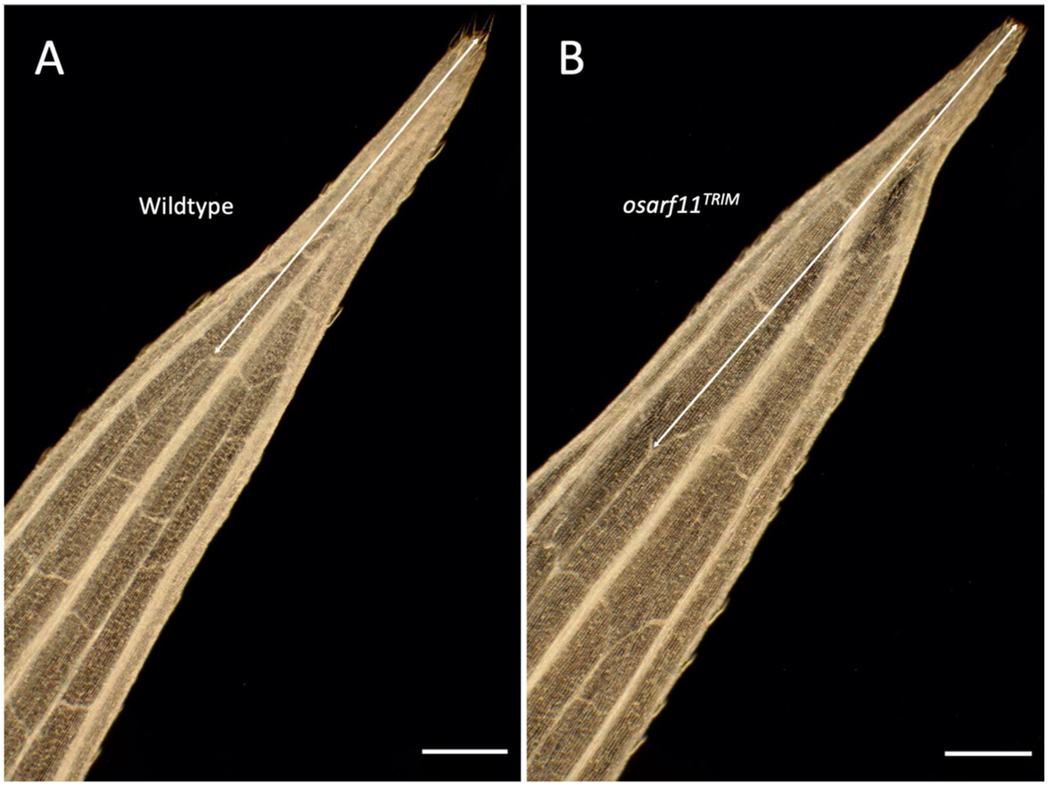

Figure S5. Tertiary vein initiation in *osarf11*^{TRIM} relative to wildtype. The point of tertiary vein initiation was measured from the apex of the leaf blade in both (A) wildtype and (B) *oasarf11*^{TRIM} seedlings. Representative images were taken of leaf four of the primary shoot at three weeks of development. Two distances were measured per leaf. Arrows indicate distance measured. Scale bar = 0.3 mm.

Table S1. Quantitative phenotypes measured in *osarf11*^{TRIM} plants.

Phenotype	Wildtype	*osarf11*^{TRIM}	Difference (%)	*P**
**Roots – one week**				
Primary root length (mm)	32.59±11.24 (113)	23.71±10.36 (99)	-27	<0.0001
Crown root number	3.03±1.39 (113)	2.24±1.34 (99)	-26	<0.0001
Lateral root number	6.05±5.47 (39)	2.18±3.26 (34)	-64	0.0006
**Blade length (mm) – two weeks**				
Cotyledon	11.04±1.24 (21)	10.17±1.37 (27)	-8	0.0255
Leaf two	11.67±1.81 (21)	9.09±1.60 (27)	-22	<0.0001
Leaf three	27.52±4.97 (21)	21.69±4.93 (27)	-21	<0.0002
Leaf four	59.21±20.99 (21)	37.70±19.67 (27)	-36	0.0005
**Blade width (mm) – two weeks**				
Leaf two	2.23±0.28 (19)	1.85±0.18 (22)	-17	<0.0001
Leaf three	2.85±0.34 (16)	2.31±0.27 (22)	-19	<0.0001
Leaf four	3.16±0.18 (15)	2.50±0.32 (8)	-21	<0.0001
**Blade width (mm) – four months**				
Tallest blade	12.9±0.19 (15)	10.75±0.78 (14)	-17	<0.0001
**Height (mm) – two weeks**				
Total height	104.72 ±25.21 (118)	78.39±41.24 (91)	-25	<0.0001
First internode	19.49±2.15 (21)	18.46±2.21 (27)	-5	0.1115
Second internode	23.81±3.46 (21)	23.16±4.13 (27)	-3	0.5630
**Height (mm) – four months**				
Total height	92.69±4.76 (15)	95.61±7.19 (14)	+3	0.2061
**Biomass (g) – two weeks**				
Total biomass	0.16±0.04 (118)	0.10±0.05 (91)	-38	<0.0001
Shoot	0.12±0.03 (16)	0.08±0.03 (14)	-33	0.0005
Root	0.055±0.014 (16)	0.036±0.013 (14)	-35	0.0004
**Dry Biomass (g) – six months**				
Total biomass	56.18±9.10 (10)	52.02±10.43 (10)	-7	0.1772
Shoot	32.29±4.19 (10)	35.99±5.54 (10)	+11	0.1104
Root	23.89±7.65 (10)	16.03±6.72 (10)	-33	0.0253
**Panicles**				
Length of panicle axis (mm)	154.34±27.75 (84)	151.89±21.19 (103)	-2	0.5247
Number of panicle branches	10.0±2.14 (84)	8.39±1.51 (103)	-16	<0.0001
Panicle branch density	0.065±0.009 (84)	0.055±0.007 (103)	-15	<0.0001
**Seeds**				
Total grains per panicle	95.16±25.49 (49)	83.57±23.52 (56)	-12	0.0172
Filled grains per panicle	49±24.72 (49)	27±16.43 (56)	-45	<0.0001
Weight (g)	0.018±0.003 (100)	0.015±0.003(100)	-17	<0.0001
Length (mm)	4.97±0.16 (100)	4.88±0.21 (100)	-2	0.0005
Width (mm)	3.08±0.13 (100)	2.8±0.18 (100)	-9	<0.0001
Thickness (mm)	1.98±0.16 (100)	1.75±0.19 (100)	-12	<0.0001

Morphological traits evaluated in *osarf11*^{TRIM} relative to wildtype control plants. Shoot and root phenotypes were measured at different time periods in independent growth trials. Panicle length was measured from the first node to the tip of the panicle. Panicle branch measurements consist of the number of lateral branches of the main axis of the panicle; branch density = number of panicle branches/panicle length. Seeds selected for measurements were

harvested by starting from the tip of the panicle (ten panicles from ten plants per genotype) to remove positional bias on seed filling. Measurements were taken by hand using an electronic caliper. *P*-values were calculated in JMP using a Student's T-test. Difference (%) = (*osarf11^TRIM^* –wildtype)/(wildtype)*100. Mean ± standard deviation (sample size).

Table S2. Leaf venation defects in *OsARF11^TRIM^* seedlings.

Phenotype	Wildtype	*osarf11^TRIM^*	Difference (%)	*P**
**LV number – two weeks**				
Leaf two	15.53±2.29 (19)	12.77±1.66 (22)	-18	<0.0001
Leaf three	15.81±2.51 (16)	11.95±1.65 (22)	-24	<0.0001
Leaf four	19.47±1.3 (15)	14±2 (8)	-28	<0.0001
**LV number – three weeks**				
Leaf two	16.70±0.88 (23)	13.88±2.20 (17)	-17	<0.0001
Leaf three	17.32±2.63 (25)	13.72±2.42 (18)	-21	<0.0001
Leaf four	19.68±1.97 (25)	16.42±2.87 (19)	-17	0.0002
**LV density (veins/mm) - two weeks**				
Leaf two	6.97±0.75 (19)	6.88±0.61 (22)	-1	0.6798
Leaf three	5.54±0.44 (16)	5.18±0.32 (22)	-7	0.0061
Leaf four	6.18±0.36 (15)	5.62±0.16 (8)	-9	0.0092
**LV density (veins/mm) – three weeks**				
Leaf two	6.88±0.59 (40)	6.83±0.93 (32)	-0.7	0.796
Leaf three	5.73±0.46 (42)	5.46±0.49 (31)	-5	0.0178
Leaf four	6.09±0.59 (39)	5.74±0.67 (30)	-6	0.0198
**CV in leaf four – two weeks**				
Vein density (veins/mm^2)	7.62±1.0 (16)	6.73±0.89 (10)	-12	0.0316
Distance between two CVs (mm)	0.77±0.11 (16)	0.70±0.13 (13)	-9	0.1377
Area enclosed by two CVs and LVs (mm^2)	0.086±0.02 (16)	0.109±0.02 (13)	+27	0.0072
**CV in leaf four – three weeks**				
Vein density (veins/mm)	7.79±1.16 (24)	6.64±1.13 (18)	-15	0.0026
Distance between two CVs (mm)	0.69±0.12 (25)	0.75±0.09 (17)	+9	0.1129
Area enclosed by two CVs and LVs (mm^2)	0.091±0.02 (25)	0.125±0.03 (18)	+37	<0.0001

Vasculature was evaluated in independent populations of two and three-week-old wildtype and *osarf11^TRIM^* seedlings. Measurements were taken at the widest part of the leaf. Each data point representing the distance between commissural veins and the area enclosed by commissural veins is an average of measurements across the width of the leaf. P-values were calculated using a Student's T-test. Difference (%) = (osarf11^TRIM^ – wildtype)/(wildtype)*100. LV, longitudinal veins; CV, commissural veins. Leaf vein density = number of veins per mm leaf width. Mean ± standard deviation (sample size).

Table S3. Quantitative phenotypes observed in *osarf11*$^{TOS\text{-}17}$ plants.

Phenotype	Wildtype	*osarf11*^{TOS17}	Difference (%)	*P**
**Roots – three weeks**				
Primary root length (mm)	52.06±10.89 (16)	35.94±16.67 (18)	-31	0.0024
Crown root number	11.88±1.71 (16)	10.44±3.12 (18)	-12	0.1141
Lateral root number	168.00±22.57 (16)	88.83±52.66 (18)	-47	<0.0001
**Height (mm)**				
Three months	983.64±102.3 (11)	837.86±22.25 (14)	-15	<0.0001
**Leaf width (mm)**				
Primary shoot – Leaf two	2.62±0.34 (7)	2.15±0.25 (14)	-18	0.0096
Primary shoot – Leaf three	3.26±0.11 (5)	2.79±0.34 (5)	-14	0.0197
Primary shoot – Leaf four	4.62±0.6 (5)	3.43±0.45 (5)	-26	0.0077
**LV number**				
Leaf two	14.29±1.11(7)	11.64±1.65 (14)	-19	0.0012
Leaf three	21±1 (5)	17±2.24 (5)	-19	0.0065
Leaf four	24.4±2.19 (5)	20.4±1.52 (5)	-16	0.01
**LV density (veins/mm)**				
Primary shoot – Leaf two	5.51±0.61 (7)	5.41±0.62 (14)	-2	0.7368
Primary shoot – Leaf three	6.45±0.34 (5)	6.08±0.28 (5)	-6	0.0992
**Seeds**				
Filled grains		**Complete sterility*		

Morphological traits evaluated in *osarf11*$^{TOS\text{-}17}$, which overlap with traits assessed in *osarf11*^{TRIM}, next to its wildtype counterpart. Venation measurements were taken at the widest part of the leaf. P-values were calculated using a Student's T-test. Difference (%) = (*osarf11*^{TRIM} – wildtype)/(wildtype)*100. Leaf vein density = number of veins per mm leaf width; LV = longitudinal veins. Mean ± standard deviation (sample size).
